# Establishment of a predictive model for spontaneous preterm birth in primiparas with grade A1 gestational diabetes mellitus

**DOI:** 10.3389/fgwh.2025.1496085

**Published:** 2025-03-06

**Authors:** Ting Sun, Yangyang Zhang, Chunzhi Xie, Anyi Teng, Shi Lin, Hui Zhang, Yan Li

**Affiliations:** ^1^ Department of Gynaecology and Obstetrics, Maternal and Child Health Hospital, Shanghai, China; ^2^ Department of Gynaecology and Obstetrics, Xinhua Hospital Affiliated to Shanghai Jiao Tong University School of Medicine, Shanghai, China

**Keywords:** gestational diabetes mellitus, grade A1, spontaneous preterm birth, prediction model, random forest model

## Abstract

**Objective:**

To establish a predictive model for spontaneous preterm birth (SPB) in primiparas with grade A1 gestational diabetes mellitus (GDM).

**Methods:**

The clinical data of 1,229 primiparas with grade A1 GDM who delivered in our hospital from July 2020 to August 2023 were retrospectively analyzed, including 142 primiparas in the SPB group and 1,087 primiparas in the full-term group. Their basic information, family history, weight, cervical length (CL) measured by transvaginal ultrasound in the second trimester, and pregnancy complications were analyzed. The factors influencing SPB were explored, and a prediction model based on a random forest algorithm was constructed.

**Results:**

Short CL in the second trimester, a family history of preterm birth, a high pre-pregnancy and prenatal body mass index, the use of assisted reproductive technology, and a high fasting blood glucose level in the first trimester were important risk factors for SPB in primiparas with grade A1 GDM. The prediction model constructed in this study has a high overall prediction angle.

**Conclusions:**

Evaluation of the above risk factors before or during pregnancy and preventive measures and interventions targeting these risk factors will reduce the risk of SPB in primiparas with grade A1 GDM.

## 1 Introduction

Preterm birth (PB) is defined as delivery before 37 weeks of gestation. Different countries have different definitions of the lower limit of PB based on differences in their neonatal care capacity. In the United States, it is 20 weeks, while it is 28 weeks in China (1, 2). PB is divided into spontaneous preterm birth (SPB), preterm premature rupture of membranes (PROM), and iatrogenic preterm birth, among which SPB accounts for approximately 50% of cases. However, there were still 13.4 million preterm infants worldwide in 2020, accounting for more than 1 in 10 births (3). Preterm birth complications are the leading cause of death in children under 5 years of age, and preterm birth was responsible for approximately 900,000 deaths among children in 2019 (4). Many surviving preterm infants may have long-term disabilities, including cerebral palsy, visual or hearing impairment, delayed social development, increased behavioral problems, and an increased risk of chronic disease in adulthood (5, 6). Therefore, prevention of preterm birth is key to reducing the risk of neonatal morbidity and mortality. Studies have found that SPB occurs in approximately 5% of primiparas (7). At present, the main predictor used in the prediction models of SPB at home and abroad are cervical length (CL) measured by transvaginal ultrasound, fetal fibronectin (fFN), phosphorylated insulin-like growth factor binding protein 1 (phIGFBP-1), complement C5 in cervical secretions, and cell-free fetal DNA in maternal peripheral blood (8, 9). However, some studies have found that the positive predictive value of CL and fFN for SPB in primiparas is low (7). Moreover, the economic costs of using fFN and other serological tests as routine SPB screening tools for primiparas are high. Therefore, further improvement of prediction models is needed to improve their predictive value.

Gestational diabetes mellitus (GDM) is a type of diabetes that occurs or is first diagnosed during pregnancy, with an incidence rate of 2.3%–29.6%. Grade A1 GDM was defined as those whose blood glucose could be well controlled by nutritional management and exercise guidance. Grade A1 GDM accounts for a relatively high proportion of GDM. It may be due to placental vascular lesions or inflammatory reactions and other reasons, the risk of PB in pregnant women with grade A1 GDM is 1.4 times higher than that in pregnant women with normal blood glucose levels (10). There is currently no prediction model for the occurrence of SPB in primiparas with grade A1 GDM. Random forest model is a conventional machine learning tool that can process large data sets, assess the importance of variables on the prediction of dependent variables, generate accurate and simple results, and provide reference for subsequent clinical decision making (11–13).We explored the risk factors for SPB in primiparas with grade A1 GDM using a retrospective cohort study design and established a prediction model using a random forest algorithm. The results of this study will provide scientific evidence for early warning and intervention for SPB in primiparas with grade A1 GDM.

## 2 Materials and methods

### 2.1 Participating cohorts and inclusion criteria

Pregnant women managed in the Maternal and Child Health Hospital, Songjiang, Shanghai, China, from July 2020 to August 2023 were included in this study. The inclusion criteria included a diagnosis of grade A1 GDM (14), primiparity, normal placental position. The exclusion criteria included multiple pregnancies, cervical insufficiency, and indicated preterm birth, incomplete data. The study protocol was approved by the ethics committee of the hospital (Ethical approval number: 20210201), and all participants signed a written informed consent form before enrolment in the study. A total of 1,229 participants were included in the analysis. There were 142 cases (11.55%) in the SPB group (who delivered at a gestational age of between ≥28 weeks and <37 weeks), and 1,087 cases (88.45%) in the full-term birth (FTB) group (who delivered at a gestational age of ≥37 weeks).

### 2.2 Definition of terms

#### 2.2.1 Gestational age

It is calculated from the first day of the last menstrual period, and 7 days are defined as a week of gestational age.

#### 2.2.2 Grade A1 GDM

GDM refers to the glucose metabolism that is normal before pregnancy and appears during pregnancy. 75gOGTT is performed at 24–28 weeks of gestation. The thresholds of fasting, 1 h and 2 h after glucose intake are 5.1 mmol/L, 10.0 mmol/L and 8.5 mmol/L, respectively. GDM is diagnosed when any blood glucose value reach or exceed these criteria. Grade A1 GDM is defined as those whose blood glucose could be well controlled by nutritional management and exercise guidance, that is, fasting blood glucose <5.3 mmol/L and 2-h postprandial blood glucose <6.7 mmol/L.

#### 2.2.3 Smoking in primipara or spouse

Primipara or spouse smoked during pregnancy.

#### 2.2.4 Transvaginal ultrasound method for Cl measurement

Between 20 and 24 weeks of gestation, the primipara was placed in lithotomy position after she urinates (15). After this, an ultrasound probe was placed in the vagina of the primipara and rotated to show a sagittal section of the cervix and display the internal and external opening of the cervix and the entire cervical canal. The linear distance from the internal to the external opening of the cervix was measured three consecutive times, and the smallest value was used.

#### 2.2.5 Methods of sampling for the ureaplasma urealyticum (UU) test

At 11–13 + 6 weeks of gestation, a swab was inserted into the distal 2–3 cm of the cervical canal and kept there for approximately 15 s. It was then gently rotated from side to side until it was covered with cervical secretions and removed.

#### 2.2.6 Methods of sampling for the group B streptococcus (GBS) test

At 35–36 weeks of gestation, during PB or during PPROM, samples were taken from the lower third of the vagina using a swab. Samples were concurrently obtained from the rectum through the rectal sphincter using the same swab (16).

### 2.3 Exposures and covariates

The main objective of the study was to develop a prediction model for SPB in primiparas with grade A1 GDM. We extracted information from the electronic medical record system including age, height, use of assisted reproductive technology (ART), pre-pregnancy and prenatal weight, CL measured by transvaginal ultrasound at 20–24 weeks of gestation, fasting blood glucose (FBG) levels in the first trimester, trimester in which GDM was diagnosed, prenatal hemoglobin (HB) levels, hypertensive disorders of pregnancy (HDP), subclinical hypothyroidism (SCH), intrahepatic cholestasis of pregnancy (ICP), premature rupture of membranes (PROM), ureaplasma urealyticum (UU) infection in the first trimester, Group B Streptococcus (GBS) infection in the last trimester, smoking in a primipara or her spouse (smoking index ≥200), and family history of PB and DM. All primiparas underwent screening for GDM, with a 75-g oral glucose tolerance test (OGTT) performed at 24–28 weeks of gestation. The blood glucose thresholds during fasting and one and two hours after oral glucose intake were 5.1, 10.0, and 8.5 mmol/L, respectively. GDM was diagnosed if the blood glucose values reached or exceeded these thresholds at any time point. GDM was also diagnosed if the OGTT was normal but the FBG was ≥5.1 mmol/L after 28 weeks. Patients with ideal blood glucose control after nutritional management and exercise guidance during pregnancy were considered to have grade A1 GDM.

### 2.4 Statistical analysis

#### 2.4.1 Basic methods

Epidata version 3.0 software was used for data entry, and SPSS version 22.0 statistical software was used for statistical description of all the included independent variables. Continuous variables were described using the means ± standard deviations, and categorical variables were described using the adoption rate or component ratio. After descriptive statistics, the difference between the two groups was compared. Based on the distribution of the continuous variables, differences between the groups were assessed using a *t*-test or Wilcoxon rank sum test; for categorical variables, the difference between the groups was calculated using a chi-square test or a fisher's exact test. In the comparison of the differences between the groups, a *P* of <0.05 indicated a significant difference.

#### 2.4.2 The random forest algorithm

The study predicted preterm birth using variables obtained from clinical. During the screening of variables, the predictors that were highly consistent with the predicted outcome, such as the delivery week, were first excluded. After this, postpartum-related predictors, such as the newborn weight and newborn sex, were excluded. A total of 19 variables were used to predict the occurrence of preterm birth. When constructing the random forest model, the model parameters with the least error were determined using different values of the random forest model parameters (the number of features and the number of decision trees), so as to achieve as high an accuracy of the prediction model as possible. After the model parameters were determined, the prediction model using various predictors of premature birth was constructed. In this study, the validation of the random forest model adopted two methods: internal validation and repeated fitting. The internal validation randomly divided the complete data set into two parts: the training set and the validation set. The training set was 70% and the validation set was 30%. Repeated fitting meant that ten random cross iterations were performed during model construction to evaluate the stability of the construction of the prediction model by comparing the consistency of the results of ten prediction models in the fitting effect (using AUC, accuracy, sensitivity and specificity of prediction) and important parameters. At the same time, this made it convenient to select the prediction model with the highest sensitivity while ensuring high accuracy among multiple prediction models.

## 3 Results

### 3.1 Baseline characteristics of the study population

A total of 1,282 participants were recruited in this study. After excluding those with cervical insufficiency (*N* = 7) and indicated preterm birth (*N* = 46), 1,229 primiparas were included in the final analysis, of whom 142 had PB (11.55%) and the remaining had FTB. As shown in Table 1, the baseline characteristics of the SPB and FTB groups were compared, and the results showed that there were statistically significant differences in pre-pregnancy weight, cervical length at 20–24 weeks of gestation, prenatal Hb levels, use of ART, GBS infection rates, PROM, smoking in the primiparous woman or her spouse, and family history of PB and DM between the two groups (*P* < 0.05). However, there were no statistically significant differences in other indicators (*P* > 0.05).

**Table 1:** Baseline characteristics of the study population.

Variable	Total (*n* = 1,229)	SPB group (*n* = 142)	FTB group (*n* = 1,087)	*P* value
Gestational age (weeks)	38.72 ± 1.76	34.66 ± 1.43	39.25 ± 0.88	<0.01
Age (years)	29.30 ± 3.71	29.73 ± 3.80	29.24 ± 3.69	0.150
Height (cm)	161.10 ± 5.04	160.60 ± 5.36	161.10 ± 5.00	0.268
Pre-pregnancy weight (kg)	58.59 ± 10.01	61.44 ± 10.02	58.21 ± 9.95	<0.01
Prenatal weight (kg)	70.20 ± 10.52	70.73 ± 10.89	70.13 ± 10.48	0.537
FBG in first trimester (mmol/L)	5.10 ± 0.54	5.06 ± 0.66	4.95 ± 0.53	0.056
Trimester of diagnosing GDM				0.075
In second trimester	897 (72.99%)	113 (79.58%)	784 (72.13%)	
In last trimester	332 (27.01%)	29 (20.42%)	303 (27.87%)	
CL (mm)	28.05 ± 3.38	23.60 ± 3.74	28.63 ± 2.85	<0.01
Prenatal Hb (g/L)	116.40 ± 13.11	114.60 ± 11.36	116.70 ± 13.31	0.045
ART				<0.01
Yes	77 (6.27%)	22 (15.49%)	55 (5.06%)	
No	1,152 (93.73%)	120 (84.51%)	1,032 (94.94%)	
UU infection				0.259
Yes	75 (6.10%)	13 (9.15%)	62 (5.70%)	
No	1,154 (93.90%)	129 (90.85%)	1,025 (94.30%)	
GBS infection				<0.01
Yes	69 (5.61%)	16 (11.27%)	53 (4.88%)	
No	1,160 (94.39%)	126 (88.73%)	1,034 (95.12%)	
HDP				
Yes	61 (4.15%)	11 (7.75%)	50 (4.60%)	0.156
No	1,168 (95.04%)	131 (92.25%)	1,037 (95.40%)	
SCH				0.699
Yes	106 (8.62%)	11 (7.75%)	95 (8.74%)	
No	1,123 (91.38%)	131 (92.25%)	992 (91.26%)	
ICP				0.988
Yes	22 (1.79%)	2 (1.41%)	20 (1.84%)	
No	1,207 (98.21%)	140 (98.59%)	1,067 (98.16%)	
PROM				<0.01
Yes	255 (20.75%)	74 (52.11%)	181 (16.65%)	
No	974 (79.25%)	68 (47.89%)	906 (83.35%)	
Smoking in primipara				<0.01
Yes	6 (0.49%)	5 (3.52%)	1 (0.09%)	
No	1,223 (99.51%)	137 (96.48%)	1,086 (99.91%)	
Smoking in spouse				0.104
Yes	292 (23.76%)	42 (29.58%)	250 (23.00%)	
No	937 (76.24%)	100 (70.42%)	837 (77.00%)	
Family History of PB				<0.01
Yes	19 (0.98%)	18 (12.68%)	1 (0.09%)	
No	1,210 (98.45%)	124 (87.32%)	1,086 (99.91%)	
Family History of DM				<0.01
Yes	157 (12.77%)	33 (23.24%)	124 (11.41%)	
No	1,072 (87.23%)	109 (76.76%)	963 (88.59%)	

FBG, fasting blood glucose; GDM, gestational diabetes mellitus; CL, cervical length; Hb, hemoglobin; ART, assisted reproductive technology; UU, ureaplasma urealyticum; GBS, Group B Streptococcus; HDP, hypertensive disorders of pregnancy; SCH, subclinical hypothyroidism; ICP, intrahepatic cholestasis of pregnancy; PROM, premature rupture of membranes; PB, preterm birth; DM, diabetes mellitus; SPB, spontaneous preterm birth; FTB, full-term birth.

### 3.2 Effect values and confidence intervals for variables with significant differences between groups

Without controlling for any confounding factors, we analyzed the effect on outcomes of PB with the predictor variables in Table 1 with significant between-group differences. As shown in Table 2, increased pre-pregnancy weight, ART, GBS infection, shortened CL, PROM, smoking in pregnant woman, family history of PB and DM were independent risk factors for SPB in grade A1 GDM.

**Table 2:** Effect size and confidence intervals for variables with significant differences between groups.

Variable	95% CI		
	Effect size	Lower Bound	Upper Bound
Pre-pregnancy Weight	−0.030	−0.046	−0.013
ART	1.236	0.706	1.765
GBS Infection	0.907	0.318	1.496
CL	0.432	0.370	0.494
PROM	1.695	1.329	2.061
Smoking in Pregnant Woman	3.680	1.526	5.834
Family History of PB	5.060	3.037	7.083
Family History of DM	0.855	0.423	1.287

ART, assisted reproductive technology; GBS, Group B Streptococcus; CL, cervical length; PROM, premature rupture of membranes; PB, preterm birth; DM, diabetes mellitus.

### 3.3 Importance ranking in the random forest prediction model for SPB in primiparas with grade A1 GDM

**Figure 1:**
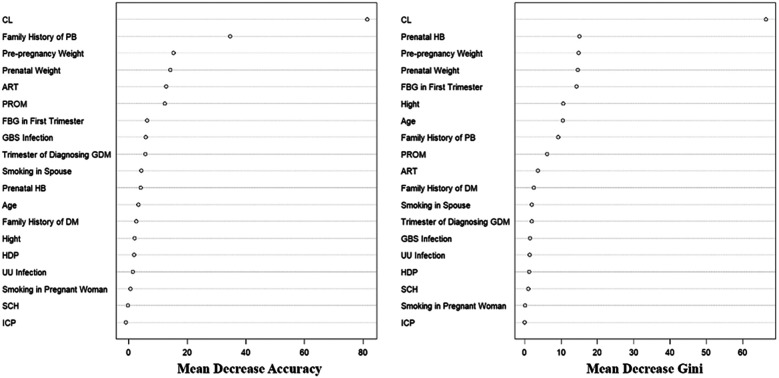
The figure of variable importance ranking in the random forest prediction model.

In the process of building the random forest model, the parameters involved mainly include ntree and mtry. Among the 1,229 included participants, the data of 70% of them were extracted by the bootstrap method to establish a random forest training model. Random forest models with different parameters were created and verified on the validation set. It was found that when mtry was 17, the error of the prediction model was the smallest. Decision trees could completely separate the predicted results. The overall misjudgment rate of the model was 6.95%. According to the average downward trend of Gini coefficient of each risk factor in the random forest model, the importance ranking of the influencing factors for SPB in primiparas with grade A1 GDM is shown in Figure 1. CL, family history of PB, pre-pregnancy weight, prenatal weight, ART, PROM, FBG in first trimester, were important in multiple prediction models.

### 3.4 ROC curve of the predictive value of the random forest model

**Figure 2:**
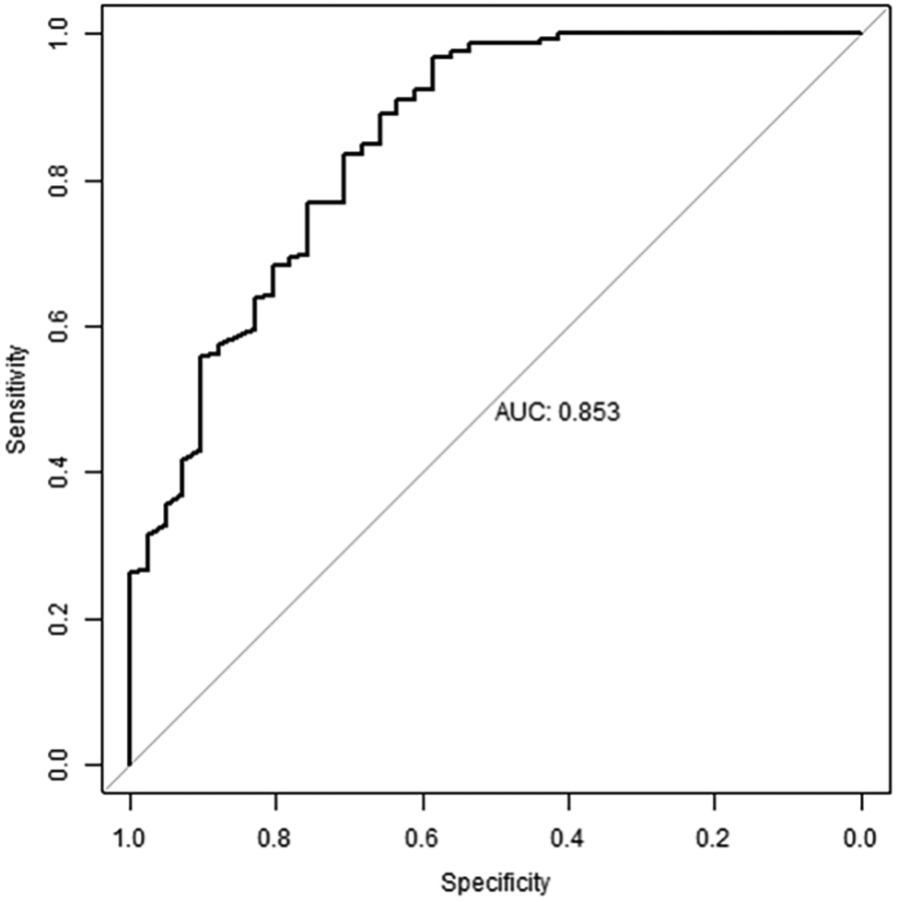
ROC Curve for random forest model.

Using the fitting results of Model 1 as an example, the AUC value of the fitted model was 0.853 (reference value was 0.8 or 0.85), the AUC value of the fitted model was 0.853 (reference value: 0.8 or 0.85), the sensitivity and specificity were 0.634 and 0.976, respectively, as shown in Figure 2.

### 3.5 The cross-validation results

The remaining nine times of cross-validation showed that the results were relatively robust, as shown in Table 3. Among the 10 prediction models randomly constructed, the minimum AUC value was 0.853 and the maximum AUC value was 0.932. The ROC curve and AUC value showed that the overall prediction Angle of the prediction model constructed in this study was high.

**Table 3:** The cross-validation results.

Model number	AUC	Sensitivity	Specificity
1	0.853	0.634	0.976
2	0.872	0.474	0.987
3	0.930	0.519	0.926
4	0.932	0.500	0.941
5	0.882	0.587	0.912
6	0.890	0.577	0.981
7	0.864	0.551	0.927
8	0.926	0.481	0.926
9	0.877	0.533	0.959
10	0.904	0.558	0.944

AUC, area under curve.

## 4 Discussion

Due to health economics and other reasons, we cannot establish a prediction model for SPB in all pregnant women. However, the incidence of SPB in GDM women is higher than that in the general population. For GDM women with serious complications, we can combine biochemical or serological tests to predict preterm birth. However, grade A1 GDM does not require drug treatment and is easily ignored by pregnant women and doctors. Therefore, it is of certain clinical significance to establish a prediction model based on baseline characteristics and some parameters in routine examination during pregnancy. Currently, there is a lack of classification of GDM levels in research on prediction models for SPB in primiparas at both domestic and international levels, and there is currently no predictive model for grade A1 GDM in primiparas. In recent years, random forest algorithm has been widely used in disease risk prediction, early warning and prognosis, and the prediction accuracy is also accurate. However, there are few studies on the risk prediction of SPB in primiparas with GDM by random forest algorithm. This study used a retrospective cohort design and the random forest algorithm to determine the risk factors associated with SPB in primiparas diagnosed with grade A1 GDM. The findings revealed a significant association between SPB and certain factors present during the second trimester in primiparas with grade A1 GDM, including a short CL, family history of PB, high pre-pregnancy and prenatal BMI, ART, and FBG during the first trimester. Assessing these factors before or during pregnancy and directing interventions towards high-risk individuals identified using this predictive model has paramount importance in the prevention of SPB in primiparas with grade A1 GDM.

This study found a significant association between a short CL during the second trimester of pregnancy and SPB. Currently, predicting PB in clinical practice heavily relies on transvaginal ultrasound measurement of the CL. The 2021 guidelines of the American College of Obstetricians and Gynecologists recommend that pregnant women without a history of PB undergo CL measurement using transabdominal or transvaginal ultrasound during the second trimester fetal anomaly screening (1). However, due to the absence of a standardized cut-off value for the CL, controversy over the use of CL for PB screening persists. In a prospective cohort study of 9,410 primiparas with singleton pregnancies conducted by Esplin et al. (7), it was observed that CL shortening exhibited a modest positive predictive value of only 15.5% for SPB. Although the sensitivity and predictive value of CL in predicting SPB may vary, it remains one of the predictive factors for effective interventions such as cervical cerclage insertion, cervical support, and administration of progesterone preparations. Therefore, measuring the CL during the second trimester can play a role in predicting and preventing PB.

There has been little research on the relationship between family history of PB and SPB. In a retrospective study of 23,816 pregnant women, women whose mothers had a history of PB had a 1.44 times higher risk of PB than other women, and those whose sisters had a history of PB had a 2.25 times higher risk of PB than other women (17). According to Yehonatan Sherf's research (18), after controlling for parity, gestational age, and pre-eclampsia, the presence of a maternal history of PB increased the risk of PB in pregnant women by 34%. Our findings aligned with those of the aforementioned study. This may be explained by a genetic predisposition to PB; however, further investigation is required to elucidate the underlying mechanisms.

We also observed that higher pre-pregnancy and prenatal BMI were significantly associated with SPB, which is consistent with the majority of previous research findings (19, 20). The pathogenesis of the association between high BMI and SPB remains unclear and is possibly related to an abnormal expression of cytokines in overweight or obese pregnant women and placental transcription (21, 22). With the development in society, significant transformations have occurred in people's dietary patterns and work-life routines, presenting formidable challenges to weight management during pregnancy. Consequently, there has been an increasing prevalence of pre-pregnancy and prenatal overweight or obesity among women. Nevertheless, weight management is possible. Obstetricians can provide pertinent information and guidance to women during preconception check-ups or during their initial obstetric visit to enable them to promptly and effectively regulate their weight before and throughout pregnancy, thereby mitigating the risk of SPB.

In this study, it was discovered that ART was a risk factor for the occurrence of SPB in primiparas with grade A1 GDM. Wang et al. (23) discovered that pregnant women who undergo ART had a higher likelihood of experiencing PB than those who conceived naturally. This might be attributed to increased levels of HSP70 and NF-κB expression in the placental tissues of offspring conceived using ART. While ART brings promising advancements for individuals experiencing infertility, it also presents challenges in terms of pregnancy outcomes. Clinicians should strictly adhere to the indications for ART and provide comprehensive prenatal care prior to its implementation.

Furthermore, this study revealed that the risk of SPB increased with increasing FBG levels during the first trimester in primiparas with grade A1 GDM. A study by Chen et al. (24) found that as the FBG levels increased in the first trimester, the rate of PB also increased, especially for those with a FPG level of ≥5.6 mmol/L and abnormal OGTT who had a higher risk of PB. Due to the decrease in FBG levels during pregnancy as the gestational age increases before 24 weeks, obstetricians face uncertainties regarding the optimal timing for early intervention in blood glucose levels during early pregnancy. Diet and exercise interventions serve as the primary cornerstone for managing grade A1 GDM, necessitating the collaborative efforts of obstetricians, pregnant women, and their families to achieve blood glucose control.

In comparison to other PB prediction models, the inclusion of age and smoking history in this study did not yield statistically significant predictive effects. We speculate that the differences in our research findings may be partially attributable to variations in population characteristics, GDM screening methods, and management approaches for PB.

This model has good predictive ability, especially high specificity. It can reliably identify primiparas with low risk of SPB, avoid unnecessary medical intervention, and reduce the psychological stress of primiparas.

The pathogenesis of SPB includes cervical insufficiency, decreased progesterone action, excessive and abnormal uterine distension, vascular disorders, disruption of maternal-fetal tolerance, and induction of allergic mechanisms (25). Due to the various etiologies and pathogenic mechanisms, it is difficult for a single predictive factor to be an effective predictor (26). This study did not use a single variable, such as CL, as the only predictor of preterm birth, but combined multiple clinical characteristics as risk factors to establish a prediction model. fFN and other screening methods were not used to prevent unnecessary medical consumption. For example, pre-pregnancy obesity is a risk factor for assisted reproductive technology and short cervix. Pre-pregnancy obesity may be combined with insulin resistance and lead to increased blood glucose in early pregnancy. Therefore, the combined effect of multiple factors has a greater impact on the risk of preterm birth. The prediction model was based on the random forest algorithm, which utilized available data to construct a random forest model for SPB in primiparas with grade A1 GDM. The random forest model was built by iteratively searching for the optimal parameters and combining the parameters with the best fitting effect. The ROC curve and AUC value demonstrated that the prediction model constructed in this study had a high overall prediction angle. This predictive model aimed to aid the provision of timely and effective preventive measures for primiparas with grade A1 GDM with a high risk of SPB, with the ultimate goal of reducing the incidence of SPB.

### 4.1 Strengths and limitations

The current study had certain limitations. First, this study did not incorporate serological testing and other examinations. However, considering the high cost of such tests and their greater negative predictive value, using them for routine screening might lead to inefficient allocation of healthcare resources. Secondly, a 10-fold cross validation method was used for internal validation of the model, and no multi-center external validation was performed. The results of the study were limited to regions, populations, and medical conditions. Thirdly, this study was retrospective and had a small sample size. In the future, it is necessary to collect multi-center and different population data to verify the results of the model, which can improve the reliability of the results of the model, and other potential influencing factors that may play a role in a wider range may be mined to further improve the model for further clinical promotion.

## 5 Conclusion

In summary, our analysis using the random forest algorithm revealed that a short CL in the second trimester, a family history of PB, high pre-pregnancy and prenatal BMI, ART, and high FBG levels in the first trimester were important risk factors for SPB in primiparas with grade A1 GDM. The prediction model constructed in this study had a high overall prediction angle. The implementation of early preventive measures by obstetricians is crucial for mitigating risk factors and reducing the incidence of premature birth.

## Data Availability

The original contributions presented in the study are included in the article/Supplementary Material, further inquiries can be directed to the corresponding author.
