# Supplementary material for: Establishment of a predictive model for spontaneous preterm birth in primiparas with grade A1 gestational diabetes mellitus
Source: Front Glob Womens Health. 2025 Mar 6;6:1496085. doi: 10.3389/fgwh.2025.1496085 (PMC11922705; doi:10.3389/fgwh.2025.1496085)
Supplement: Supplementary file 1 [file Table1.docx]

Supplementary Files

- **Diagnosis of gestational diabetes mellitus(GDM)**

For all pregnant women who have not been diagnosed with pregestational diabetes mellitus, 75g oral glucose tolerance test (OGTT) at 24 to 28 weeks of gestation. Diagnostic criteria for 75g OGTT: 5.1mmol/L, 10.0mmol/L, 8.5mmol/L glucose thresholds for fasting and 1 and 2 hours after sugar, respectively. GDM is diagnosed if any blood glucose value meeting or exceeding the above criteria.

- Classification of GDM

After medical nutrition therapy and exercise guidance, the blood glucose can be controlled to the target ,that the fasting glucose <5.3 mmol/L and 2-hour postprandial glucose <6.7mmol / L,as grade A1 GDM; Pregnant women who required the addition of antidiabetic drugs to achieve glycemic control are classified as grade A2 GDM.
